# Co-production of research derived actionable resources for the delivery of Individual Service Funds (EQUALD study): Protocol

**DOI:** 10.1371/journal.pone.0306522

**Published:** 2024-07-03

**Authors:** Alice Dunning, Clare Tarling, Liz Croot

**Affiliations:** Division of Population Health, University of Sheffield, Sheffield, United Kingdom; Public Library of Science, UNITED STATES

## Abstract

Adults with a learning disability who receive social care are legally entitled to a personal budget. Personal budgets were introduced to promote choice and control in support. Individual Service Funds were introduced as a flexible way that personal budgets can be managed by a provider while maintaining choice and control for the individual. Individual Service Funds have been shown to improve quality of life for individuals and efficiency in support. Despite this, only 20% of local authorities offer them to adults with a learning disability, demonstrating the need for resources to be developed to support their delivery. This protocol described a co-production study with key stakeholders to develop and refine Individual Service Fund resources. Our primary aim is to co-produce two actionable resources: to support development, delivery, consistency, and sustained provision of ISFs; and to support uptake and optimal use of ISFs by recipients. We also aim to user-test and evaluate these resources with three Local Authorities. The result of this study will be two resources that will support the uptake of Individual Service Funds for adults with a learning disability that will be freely available online.

## Introduction

### Background

In the United Kingdom there are approximately 1.1 million adults with a learning disability [1]. In 2022/23 42% of Local Authorities spending in England was for adult social care where a learning disability was cited as the main reason for support, costing £6.7 billion [2]. A learning disability is defined as a reduced capacity to understand new or complicated information, learn new skills and a reduced ability to live independently [3].

The Care Act [4] requires all adults with a learning disability who receive social care are allocated a personal budget. A personal budget is the amount of money that a Local Authority agrees is needed to pay for an individual’s support, following a care needs assessment [5]. Personal budgets were introduced as a way for service users to have choice and control over services they receive. Despite this, there are concerns that Local Authorities are not offering genuine choice and control within personal budgets [6]. The majority of personal budgets are council managed. This means that often the individual does not know how much has been allocated to their budget or have choice over how this is spent. Direct payments were introduced as part of the personalisation movement in English social care policy. Direct payments provide choice of support as the individual is paid the allocated personal budget directly from the Local Authority in lieu of support, they can then control how this is spent [5]. Despite the choice and control that direct payments offer, they are not always feasible or accessible for adults with a learning disability as managing money and becoming employers of care staff is a significant legal responsibility challenge [7–9]. This means adults with a learning disability receiving direct payments rely heavily on informal carers to manage the budget [10, 11]. A further difficulty with direct payments is that some Local Authorities have viewed them as unsuitable for adults with a learning disabilitydue to a perceived lack of capacity to manage the budget [12], meaning that their offer can be restricted.

Individual Service Funds (ISFs) were outlined in the Care Act [4] as a way for an individual to have flexibility and control over their personal budget without the burden of managing a Direct payment. In an ISF, a provider organisation holds and manages the individual’s budget on their behalf [13]. Within an ISF it is vital that the individual’s personal budget is restricted for the individual’s support only, and that there is transparency over how the budget is accounted for. The individual should be empowered to choose how they want to receive support including the flexibility to spend or save [14]. The ISF also allows an individual to pay for support from providers other than their ISF provider, allowing them access to *non-traditional* support using community assets or resources [13].

ISFs were first introduced in Scotland in the late 1990s [15] forming their self-directed support ‘option 2’ and have since been introduced in England. Where ISFs are used, they have led to a significant reduction in the cost of support [16, 17], and improvements in efficiencies [18, 19]. ISFs have also improved outcomes for individuals, as adults with a learning disability who receive an ISF have reported significant improvements in their quality of life and progress towards the outcomes they are seeking to achieve (Animate, 2014).

However, despite these promising outcomes and the policy imperative to develop ISFs as the preferred option for managing personal budgets in the Care Act (2014) ten years ago, they are still not widely offered to adults with a learning disability. Only 31 English Local Authorities (20%) reported having a full ISF offer in social care available in 2023 [20] which signifies the systemic barriers and challenges to implementing ISFs in practice for this population. Therefore, there is the need for guidance or resources to be developed to support both those working in or with Local Authorities to offer ISFs, and for individuals with a learning disability and their families to understand ISFs and make a meaningful choice.

### Rationale

ISFs are situated within the complex UK social care system that relies on interactions between many stakeholders: Local Authority commissioners, other Local Authority staff (i.e., procurement, legal), Social Workers, Support providers, adults with a learning disability and family carers; therefore, using an approach that brings together multiple perspectives, such as co-production, is important to address the problem. Co-production is defined as a shared approach to decision making that enables collaboration [21]. Co-production research is a collaborative model which brings together multiple knowledge users’ perspectives to produce findings or resources that are relevant, useful, feasible, implementable and responsive to the knowledge users needs [22].

Adults with a learning disability have historically been excluded from research and their voices ignored [23]. Despite the shift to research being done *with* adults with a learning disability rather than *on* them, recognising their lived experience and the valuable contribution they can add to research, tokenistic, one- off tick box approaches to involvement often happens [24–26]. Co-production encourages equal participation between the researchers and knowledge users and therefore balances the power and ensures the voice of adults with a learning disability is heard [27, 28].

### EQUALD project

The EQUALD project is a National Institute for Health Research funded programme of research made of five work packages. The research programme has two key objectives, the first is to understand how important mechanisms operate in the context of social care systems to support or hinder the offer, uptake, and sustained provision of successful ISFs for adults with learning disabilities. The second objective is to co-produce and carry out a formative evaluation of actionable resources to support the delivery of ISFs for adults with a learning disability. To answer the first objective, the research produced a programme theory to explain how ISFs are thought to produce successful outcomes and explored different stakeholder experiences of developing, delivering, or receiving an ISF to understand how this theory might work in practice. This earlier work will influence the next co-production and formative evaluation study as it has outlined the *key ingredients* that need to work together and highlighted some of the challenges or barriers to implementing ISFs. This protocol outlines the planned study to address the second objective of the wider EQUALD project.

The outputs from this study will include two co-produced research-derived actionable resources to support the use of ISFs. One will support local authorities and support provider organisations in the development, provision, and governance of ISFs. The second will support adults with a learning disability and their allies to make decisions about whether to take up the offer of an ISF and how to get the most from an ISF.

### Objectives

1. To co-produce two actionable resources: to support development, delivery, and sustained provision of ISFs; and to support uptake and optimal use of ISFs by recipients.
2. To carry out a formative evaluation to inform further development of these resources by using them in three local authorities not yet offering ISFs.

## Methods

Ethical approval was granted by the University of Sheffield, School of Health and Related Research ethics committee (Date: 23/04/2024; REF: 059545). The participants will be providing written or verbal consent to the study. If verbal consent is taken this will be audio-recorded.

**Fig 1:**
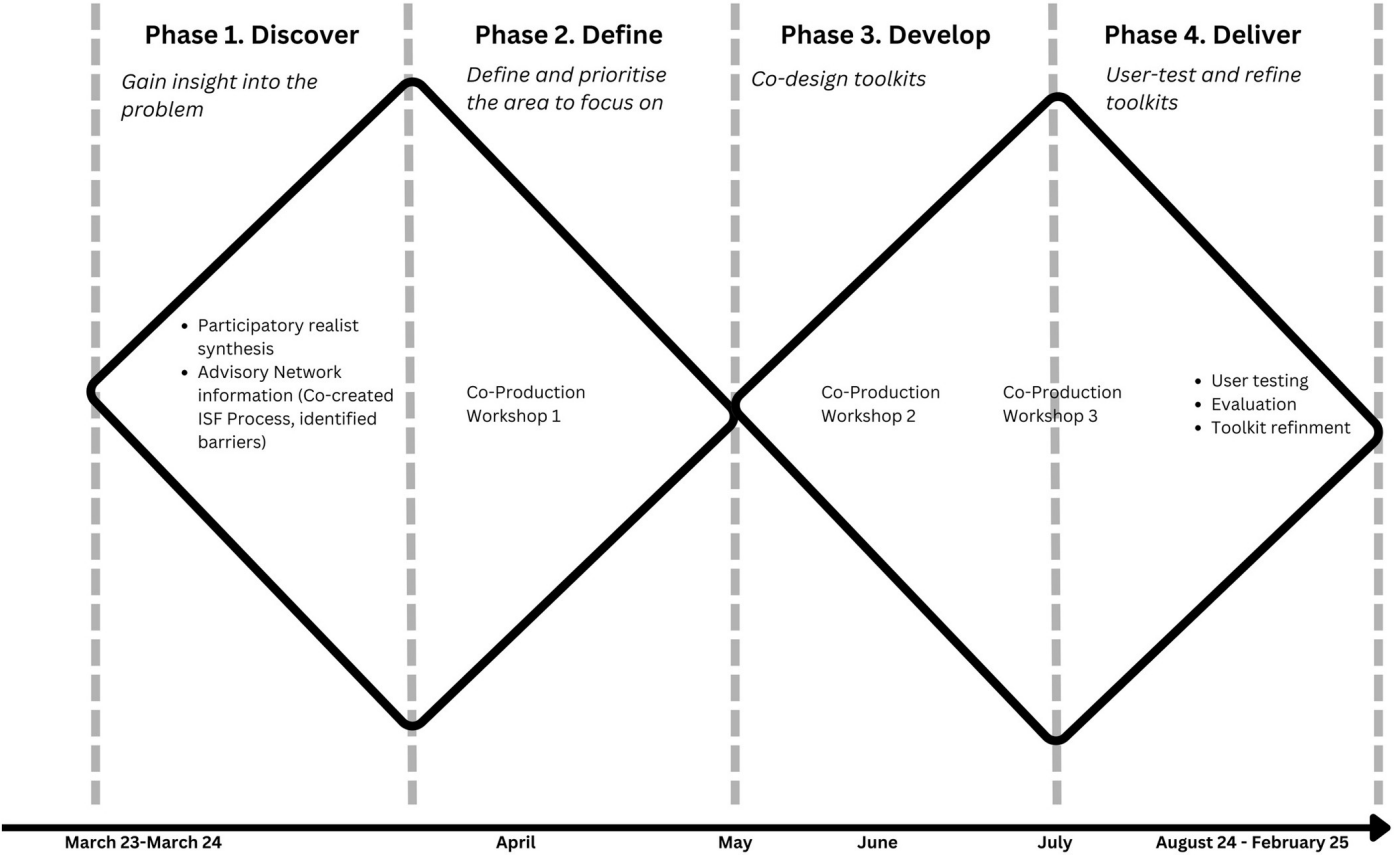
Timeline of phases based on the Double Diamond model.

The co-production work will be based upon the Design Council’s Double Diamond model [29]: Discover, Define, Develop and Deliver. Our co-production work will be mapped onto these phases. Phase one: Discover, is underway, and has involved a participatory realist synthesis and stakeholder engagement from our Advisory networks (including workshops) to identify the challenges and barriers to developing or using an ISF offer. In phase two: Define, we will work with participants in one co- production workshop and further clustered activities (small workshops, advisory meetings) to define specific problems and prioritise areas that are amenable to change using resources that could be included in the resources. Phase three: Develop, will involve iterative development of prototype resources with feedback and further development taking place across two co-production workshops and clustered activities. Phase four: Deliver, will involve formative evaluation of the tools in real world settings to further refine and develop the resources (see Fig 1).

### Patient and public involvement

The EQUALD project has included adults with a learning disability and carers throughout. There is a Project Working Group, made up of a group of self advocates who have mild to moderate learning disabilities, with or without autism who are employed by a self advocacy organisation. Individuals in this group opted to join a small working group to develop this research and the application for funding and a member of this group was a co-applicant on the funding application and now sits on the Project Management Group. The researchers (LC and AD) meet with this working group regularly to ensure the voices of group members are reflected in decisions about the management of the project. This group has contributed to the project in a number of different ways including creating our logo, reviewing and suggesting edits to our website, providing guidance on making research activities accessible, developing accessible materials, planning the co-production process and developing interview topic- guides. This self-advocacy organisation has been costed as collaborators for their work on the project. Clare Tarling, a collaborator on the project, is an expert in Easy Read design. She has ensured that all meeting agendas, presentations, meeting notes, handbooks, forms, and toolkits are all supplied in Easy Read formats. Speakup have also provided advice on accessibility and will give thorough feedback on the project outputs.

### Participants

Participants will be stakeholders who have experience of adult social care and knowledge or interest in individual service funds. Exact job titles are likely to differ in different settings but may include Local authority staff (commissioners, procurement, legal), social workers, support providers, carers and adults with learning disabilities. For the co-production phases (2,3, 4) we aim to recruit 30 participants in total ensuring we include a diverse group of service users and professionals.

#### Eligibility criteria

Professionals (Local Authority staff, social workers, support providers) who are participating must have experience of piloting, offering, or delivering ISFs, regardless of whether this was considered successful, or be in the process of setting up an ISF offer. Adults with a learning disability and carers may have experience of individual service funds, or wider self-directed support (i.e., Direct Payments) but this is not essential. Co-production workshops will take place online, so participants across England will be eligible to join. Additional research activities i.e., workshops with existing groups for adults with a learning disability or attendance at Local Authority meetings may take place face to face depending on the usual format within the setting.

### Recruitment

The co-production work will be carried out with three distinct groups of participants.

#### Discrete co-production workshops

Participants will be identified in a number of different ways. Advisory Group members and participants who were interviewed in an earlier work-package of the wider project who consented to be contacted about research opportunities will be invited to join the co-production workshops. We will work with our advisory networks to identify potential participants and promote the study to wider audiences. All those who express interest in taking part in the co-production workshops will be emailed participant information sheets and be given time to review these and ask any questions prior to participation. If participants agree to be involved, they will be asked to complete consent forms.

#### Self advocacy organisation research activities

Participants will be identified through their membership of, or attendance at, a self-advocacy organisation. They will be informed that they do not have to participate and that this will have no impact on their membership of the self-advocacy organisation. We will ask the group leads to share advertisements of workshops with adults with learning disabilities. If groups of people are willing to hear more, we will attend groups to give further information, provide Easy Read information sheets and answer any questions about the project or their involvement. If groups are happy to proceed, we will use a consent process that involved eight stages:

1. Project information sent to group leader
2. Researcher attend the group to explain more about the study
3. Researchers facilitate discussion to check the understanding and answer questions
4. Researchers assess capacity and make a decision on the capacity of individuals
5. Researchers ask if group members are willing to take part
6. Researchers remind the group of alternative choices (where possible an alternative non research activity will be offered to members who may not wish to participate in the research)
7. Group leader signs consent form to indicate the process described above has been followed
8. Members who lack capacity but who wish to engage in the study activities will be encouraged to stay in the group but their data will not be included.

#### Local Authority research activities

We have recruited three Local Authorities who are currently developing their ISF offer to be involved in the co-production phases (2, 3, 4) and the formative evaluation of the prototype resources. Participants will be identified by their Local Authority because of their role in developing ISFs. We will work with our contact at the Local Authority to share information about the research with potential participants in the best way. This might include emails, attending meetings, staff bulletins. For this, we have obtained local research governance approval to identify staff within their role at the Local Authority. Our attendance at any Local Authority meetings or co-production activities will be approved by the Local Authority and organising staff members. Prior to attending any meetings, information about the research, its purposes, the data we are collecting and how participants can opt out if they choose not to take part, will be shared. The staff member leading the meeting will send this information on behalf of the researchers. Participants will be advised to let the researchers know if they wish to join the meeting but not participate in the research. In this circumstance the researcher will not make notes of any comments these individuals make within the discussions. Group consent will be recorded for these research activities when the following steps have been completed:

1. Explain about the wider study, the goals for the co-production work
2. Provide the opportunity for any questions to be answered
3. Ask the group if they are willing to take part
4. The research team will complete a group consent form, where appropriate this will be countersigned by an individual at the Local Authority

Recruitment to all these groups will be purposeful, but we will be working with different networks to access a range of participants with different experiences of ISFs. Where necessary we will run additional research activities to allow us to respond flexibility to the needs of participants who might otherwise be excluded. For example, adults with a learning disability from ethnic minority groups, who may prefer to contribute in a smaller group with familiar people present. This responsive approach will allow us to build impact by including more participants from a broader range of backgrounds and with more diverse perspectives than would be possible in the three distinct co-production workshops alone.

### Study activities

**Fig 2:**
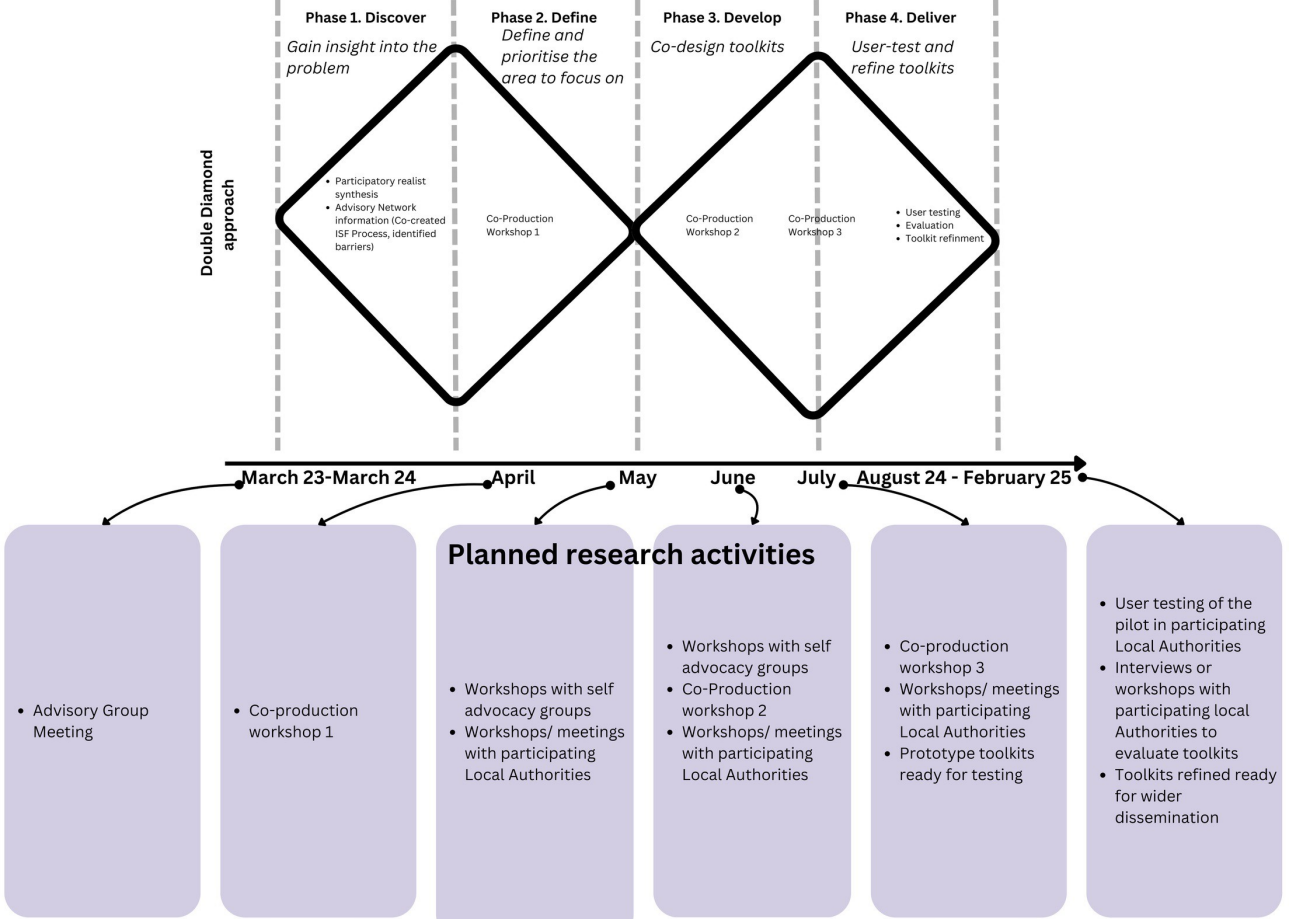
Planned research activities.

The three phases of co-production in this study (2, 3, 4) will be carried out using three discrete co-production workshops and complementary research activities with two self advocacy groups and three Local Authorities conducted in parallel (see Fig 2), as described below. The co-production research activities with the Local Authorities and self advocacy groups will feed into the three co-production workshops and vice versa in an iterative process, in this way the co-production will be cumulative. Between these research activities across the different phases the researchers will take the ideas and begin to create or draft prototype resources or materials which will be brought to future sessions. Prototypes will be continuously refined and developed in response to participants’ feedback and observations.

#### Discrete co-production workshops

Participants recruited to the discrete co-production workshops will be asked to attend all three workshops where possible to provide continuity of input. The three discrete co-production workshops will include participants from across England to help to ensure that the resources are relevant, feasible, accessible, transferable, and usable across different Local Authority settings. The workshops will be accessible for all participants. At the point of recruitment all participants will be provided with an easy-read booklet about the wider research study, explaining what we have learnt about ISFs, the process of developing and using ISFs and the key ingredients needed to make these successful. These materials were co-produced with our advisory network and will provide context and education prior to the co-production workshops. The booklet will also outline information about the purpose of the co-production work, and the expectations, priorities, and roles within the workshops. At the beginning of each discrete co-production workshop, we will discuss how we will work together. This will include setting ground rules, ensuring that everyone is included, and helping everyone to understand their role and what we aim to achieve in each session and how. Workshops will include discussions to: define the user and their needs, and to consider where the resource will be available, how it will be distributed, any promotional activities needed, as well as processes for implementation, evaluation and collection of evidence/ impact.

#### Self advocacy organisations research activities

We will run three further co-production workshops within existing meetings held by two self advocacy groups. Workshops for one group will be held online and the other will be in person, in line with the groups’ usual way of working. These workshops will focus primarily on co-producing the toolkit that will be used by adults with a learning disability and their families or carers. They will use a variety of accessible methods to promote discussion, for example, we will discuss different ways of receiving information using physical prompts such as videos, animations, and different formats of written information to support understanding.

#### Local Authority research activities

The research activities with Local Authorities will be flexible to the needs of the participating organisation or Local Authority but may include attending existing ISF planning meetings or facilitating bespoke project workshops. These activities with the Local Authorities will feed into the three co-production workshops and vice versa in an iterative process. In this way, the research activities will work in partnership with the discrete co-production workshops to co-design the resources.

#### Phase 1- Discover

The discovery phase aims to understand the context in which ISFs are used, any barriers or challenges for the delivery or uptake of ISFs and explore points where it might be possible to intervene. Much of the work of this phase has been completed already in the wider study through a realist synthesis, and engagement with our Advisory Network.

Activities within this phase will include:

1.1 Meeting with the Project Management Group to discuss the emerging challenges/ barriers within ISFs and the possible areas for change highlighted in the study’s previous work packages
1.2 Meeting with the Advisory Group to discuss the emerging challenges/ barriers within ISFs and the possible areas for change.

#### Phase 2- Define

This phase will focus on defining and prioritising the challenges or areas to focus on. During this phase we will rank the challenges in order of priority taking into account the extent to which they are amenable to change through the use of a toolkit resource, as well as ideas for mechanisms for change and possible resources to deliver these.

Activities within this phase will include:

2.1 Joining or facilitating meetings/ workshops with the participating Local Authorities
2.2 Discrete online co-production workshop 1 with a mix of stakeholders
2.3 Workshops with existing groups of adults with a learning disability
2.4 Project Management Group meeting

In the first round of co-production workshops, the focus will be to identify, prioritise and select areas that are amenable to change as well as to consider ideas for mechanisms for change, possible resources to deliver these, what format the resources might take, how they would be used and the benefits or challenges of implementing these in practice.

By the end of phase 2: Define, we will have a prioritised list of possible resources addressing specific problems or challenges in the delivery of uptake of ISFs, which are amenable to change using a resource. The researchers will be involved in these discussions and any decisions made, to ensure all options that are taken forwards are feasible within the scope of the study.

#### Phase 3- Develop

This phase will focus on prototyping and developing the resources prior to user testing. This phase will centre around discrete co-production workshops 2 and 3 and associated research activities.

Activities within this phase will include:

3.1 Discrete online co-production workshop 2 with a mix of stakeholders.
3.2 Workshops with existing groups of adults with learning disabilities.
3.3 Joining or facilitating meetings/ workshops with the participating Local Authorities.
3.4 Discrete online co-production workshop 3 with a mix of stakeholders.

In the second round of co-production workshops the focus will be on refining ideas. The researchers will bring early prototypes of several different ideas for resources so that participants have something tangible to discuss and the goal will be to prioritise two resources to be developed into full prototypes.

In the third round of co-production workshops the discussion will focus on the two prototype resources. The goal will be to further refine the prototypes and discuss their implementation. By the end of this phase, we plan to have resources ready for user-testing in Phase 4.

#### Phase 4- Deliver

This phase will include formative evaluation of the resources and further refinement.

*Formative evaluation*. The Local Authorities who are involved in the co-production of the two resources will test these in their ISF pilots. We will work with each site to identify relevant individuals to test the prototypes. The sample will include Local Authority staff and support providers who will test the first resource. adults with a learning disability and their allies will test the second resource. We cannot predict exactly how many people will be offered an ISF at each site so we cannot be precise about the sample size for this work package. However, we aim to recruit approximately 10 people to test each resource in each site, including a minimum of 6 adults with a learning disability in total. Participants from the participatory Local Authorities will take part in focus groups or semi-structured interviews. These will cover the use, relevance, acceptability, and feasibility of the resources, including areas for improvement.

### ‘Data’ capturing

All co-production workshops will be recorded for note taking purposes and extensive field notes taken. The key conclusions from each activity will be collaboratively agreed by the groups at the end of each session. The research team will also keep a co-production journal to track the design process, the evolution of ideas and refinement of the content and design of the resources, as well as reflections on the process and methods. These will be used to map good practice and any learning that can be taken forward into the later phases of co-production and future co-production research projects.

The phase 4 focus groups and interviews will be audio recorded and transcribed.

## Results and discussion

Using co-production to develop resources for ISFs should ensure that the resources developed are useful and usable. We also anticipate that by working collaboratively with stakeholders, all parties will benefit from hearing different perspectives and sharing experiences of using ISFs.

We anticipate there may be challenges with recruitment as stakeholders working within Local Authorities, and carers are stretched for time and therefore difficult to engage. In the phase 4 formative evaluation we will be reliant on the respective Local Authority’s timelines for their ISF pilot, and their selection of adults with a learning disability to take part in their ISF pilot. However, a strength of this work is the strong relationship with members of the Advisory network developed during the wider EQUALD project. Members of this network bring a range of different stakeholder perspectives and will support recruitment efforts. Additionally, we will build close relationships with the participating Local Authorities, during the co-production phases and we anticipate this will help us address any challenges that may arise and help us to respond appropriately.

The co-production workshops for adults with a learning disability will be held with self-advocacy organisations. These organisations are self-selecting for individuals who have capacity to advocate for themselves and therefore are not representative of all adults with a learning disability. A potential limitation of the study is that the needs and experiences of adults with more profound learning disabilities may not be represented in the workshops. To mitigate this, we will be working with our Advisory network to identify carers and advocates who can represent this population and we will also explore ways to make the study accessible for individuals with more profound learning disabilities.

The implementation and evaluation of the resources will be planned within the co-production work. This will ensure the implementation is feasible and the evaluation is relevant and acceptable. The prototypes will be refined further in response to user feedback and then the resources will be freely available to all Local Authorities. We will work with organisations, including those identified by our stakeholders, to ensure the resources are easy to access and freely available online. We will work with our advisory network to disseminate and advertise the resources to maximise the impact. This may include a dedicated launch of the resources advertised through multiple platforms.
